# Supplementary material for: A pan-cancer analysis of homeobox family: expression characteristics and latent significance in prognosis and immune microenvironment
Source: Front Oncol. 2025 Feb 6;15:1521652. doi: 10.3389/fonc.2025.1521652 (PMC11840236; doi:10.3389/fonc.2025.1521652)
Supplement: Supplementary Figure 1 — Mutations of each HOX gene in 32 cancers (TCGA, PanCancer Atlas) by cBioportal. [file DataSheet1.zip › Suppl.files/raw code.zip!/code/4.survival/survivalplot.zip!/survivalplot/HOXB1_OS.SKCM.pdf]

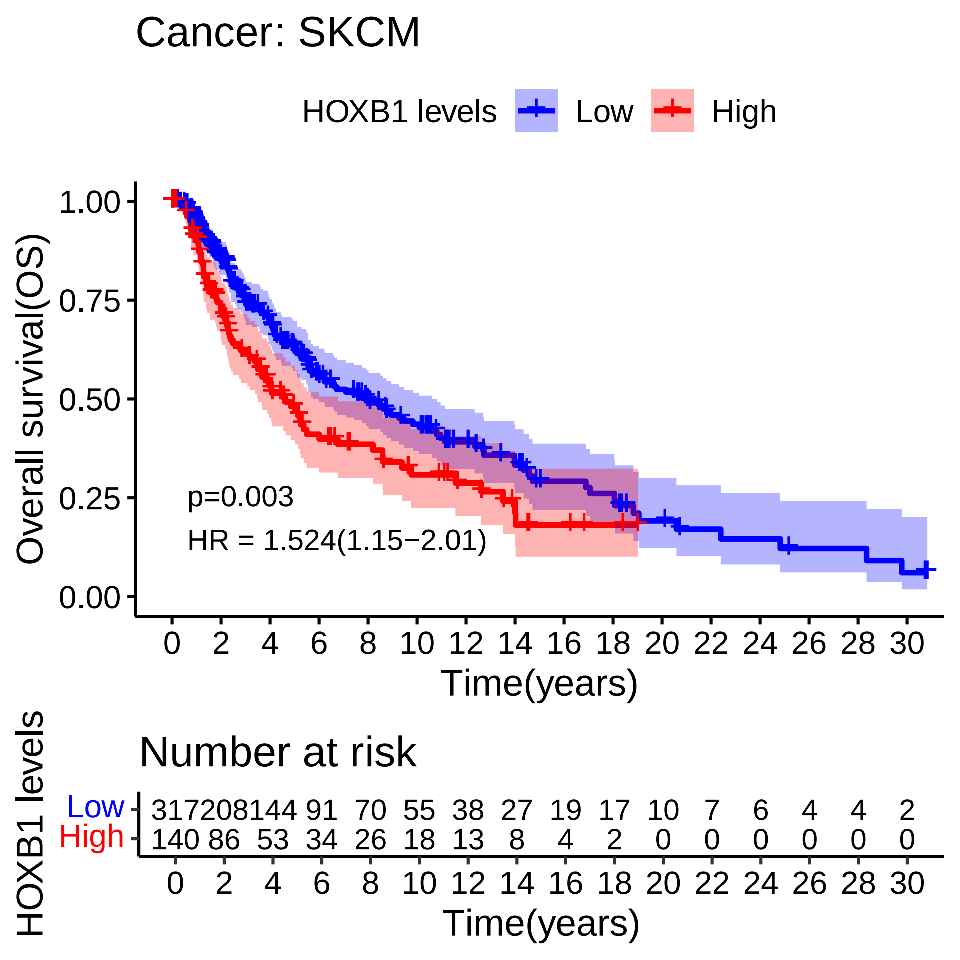
Cancer: SKCM
HOXB1 levels
Low
High
Overall survival(OS)
1.00
0.75
0.50
0.25
0.00
p=0.003
HR = 1.524(1.15−2.01)
0 2 4 6 8 10 12 14 16 18 20 22 24 26 28 30
Time(years)
Number at risk
HOXB1 levels
Low 317 208 144 91 70 55 38 27 19 17 10 7 6 4 4 2
High 140 86 53 34 26 18 13 8 4 2 0 0 0 0 0 0
0 2 4 6 8 10 12 14 16 18 20 22 24 26 28 30
Time(years)
